# High Temperature Interaction of Si-B Alloys with Graphite Crucible in Thermal Energy Storage Systems

**DOI:** 10.3390/ma13010029

**Published:** 2019-12-19

**Authors:** Jianmeng Jiao, Jafar Safarian, Bettina Grorud, Merete Tangstad

**Affiliations:** Department of Materials Science and Engineering, Norwegian University of Science and Technology (NTNU), N-7491 Trondheim, Norway; jafar.safarian@ntnu.no (J.S.); bettina_g94@hotmail.com (B.G.); merete.tangstad@ntnu.no (M.T.)

**Keywords:** Si-B alloys, graphite, PCM, carbon solubility, carbides

## Abstract

Si-B alloys are proposed as a potential phase change material (PCM) in the novel high temperature thermal energy storage systems. For successfully introducing the new PCM, the selection of proper refractory material in the PCM container is vital. At present, graphite is chosen as a potential refractory material for the PCM container, due to its high temperature stability, low thermal expansion, and high thermal conductivity. The Si-B alloys and the high-temperature interaction with graphite are hence studied. The phase formation in the Si-B alloys and the interaction with graphite at B content of 2–11 mass % and temperatures of 1450–1750 °C were investigated. Carbides were observed at the interface between the solidified alloys and the graphite. A single SiC layer was produced at B content of 2 and 3.25 mass %. Otherwise, SiC and B_4_C layers were generated at B content higher than 5 mass %. In the Si-B-C system, the phase formation is dependent on the B content. Moreover, the equilibrium B content is calculated to be 3.66 mass % in the molten Si-B alloys at 1450 °C in equilibrium with SiC and B_4_C, based on the experimental results. In this regard, the eutectic alloy (3.25 mass % B) is recommended to be used as the new PCM in the graphite container, due to that it produces simple phases and also because it is expected not to deplete any B to the B_4_C layer.

## 1. Introduction

Phase change materials (PCMs) undergo a solid–liquid phase transformation to release or absorb thermal energy at the melting/solidification process [1]. The constituent elements in the PCM play a critical role in thermal energy storage systems as it determines the energy storage ability. Then, the stored energy is transferred to the hybrid thermionic photovoltaic device which directly produces electricity based on the direct emission of electrons and photons through a vacuum space at high temperatures [2]. In order to meet the high temperature requirements for the thermionic photovoltaic converter, silicon-boron (Si-B) alloy is considered to be a potential PCM [3]. The Si and B elements have the high latent heat of fusions (1800 J/g for Si [4], 4650 J/g for B [5]), high melting temperatures (1414 °C for Si, 2092 °C for B [6]), and high thermal conductivity (142.2 W/(m∙K) for Si [7], 26 W/(m∙K) for B [8] at room temperature). However, several technical problems should be resolved before an industrial application. It includes the selection of an appropriate refractory material for the PCM container at temperatures higher than its melting point and the identification of the optimal B content in the Si-B alloys. 

In the application of the new Si-B alloys in the energy storage system at high temperatures, the selection of a compatible refractory for the PCM container is important. There is, however, a lack of reported data on the interaction of Si-B alloys with refractories. Therefore, it is critical to experimentally and theoretically investigate the interfacial reactivity between the Si-B alloys and refractory materials at high temperatures. For this purpose, two distinctly different approaches are explored by the dedicated teams, “non-wetting concept” and “passive-layer formation” [9].

The “non-wetting concept” approach was conducted by the Foundry Research Institute (FRI), aiming to find a ceramic that shows a low reactivity and non-wetting behavior in contact with the molten Si or Si-B alloys. Their published papers [9,10,11,12,13] have shown that the hexagonal boron nitride (h-BN) was the only suitable ceramic that met the requirements for PCM containers among all examined ceramic refractories. Si, Si-1B, Si-3.2B, and Si-5.7B alloys were subjected to the sessile drop experiments with h-BN substrates at temperatures up to 1750 °C [10,13]. Both the Si and Si-B alloys showed a non-wetting behavior with h-BN. Moreover, they found that the addition of B suppressed the dissolution of h-BN ceramic. 

The “passive-layer formation” approach was suggested by our group. The principle of the approach includes an in-situ reactive formation of continuous interfacial product layer at the initial stages of Si-B alloys/ceramic interaction. The formed layer would play the role of barrier coating, and thus it forms a “self-crucible” inside the PCM container. For this approach, dense graphite was selected due to its well-recognized ability to in-situ form SiC product layer in contact with molten Si. Additionally, it has the advantage of ease of machining, it is chemically stable and has a high temperature stability. It also has low thermal expansion and high coefficient thermal conductivity [14].

The interaction of Si with graphite is widely studied [15,16,17,18,19]. As soon as Si melts, a SiC layer is formed at the interface due to the chemical reaction of Si and C. The molten Si wets the graphite materials accompanied with contact angle values from 0–40° at equilibrium state. However, there is no reported data on the interaction between Si-B alloys and graphite at any temperatures.

In the Si-B system, three intermediate compounds SiB_3_, SiB_6_, and SiB_n_ have been determined [6]. SiB_3_ is also identified as SiB_4_ phase due to its homogeneous range of 52.9–58.7 mass % B [20]. SiB_6_ is regarded as a stoichiometric phase by Olesinski et al. [6]. Later, its homogeneity range is confirmed to be 68.9–70.3 mass % B [21]. Moreover, SiB_n_ corresponds to an extended solid solution between 84.4 mass % and 92.6 mass % B [6]. In the Si-rich area, Si solid solution (Si(ss)) will be in equilibrium with SiB_3_ at temperatures lower than 1270 °C and in equilibrium with SiB_6_ at 1270–1850 °C. The eutectic reaction occurs at 1385 °C at the B content of 3.25 mass %. Therefore, the B content will affect the phase formations and melting temperatures in the Si-B alloys.

**Figure 1:**
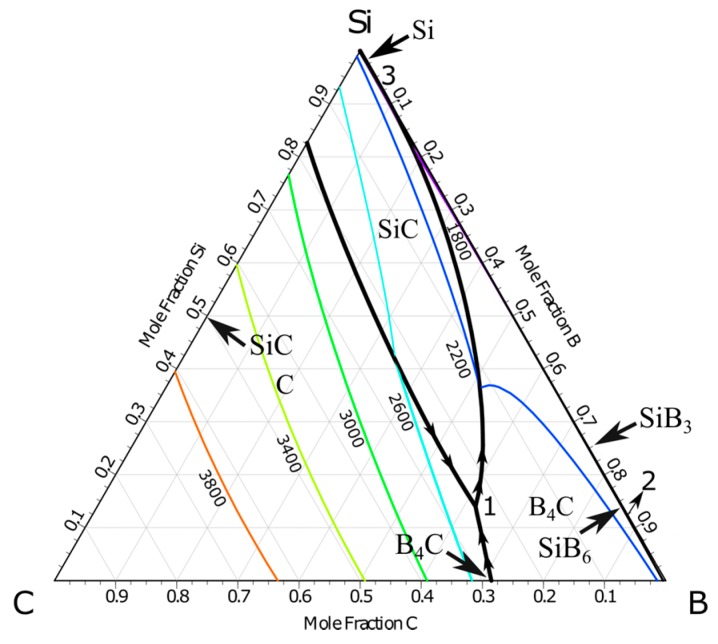
The projection of Si-B-C system at 1300–3800 °C at 1 atm (FactSage FTlite) [22].

The projection of Si-B-C system is calculated using the FactSage (Montreal, Canada and Aachen, Germany) based on FTlite commercial database [22], as shown in Figure 1. It is seen that no ternary compounds exist in the system. The stable intermediate compounds are SiC, B_4_C, and silicon borides. Of particular, B_4_C displays a wide composition between 8.8–20 at.% carbon [23]. Three four-phase intersection points with liquid alloy are found in the system. The relevant reactions in the Si-B-C system are given in Table 1 [24]. 

The purpose of this study is to investigate the high temperature interaction of Si-B alloys with graphite. Si-B alloys with 2–11 mass % B will be used in the temperature range 1450–1750 °C. The phase formations in the Si-B alloys and the interaction between Si-B alloys and graphite will be studied theoretically and experimentally during solidification. Moreover, the C solubility will be investigated in the liquid Si-B alloys at B content lower than 5 mass % and temperatures of 1450–1750 °C, aiming to calculate the equilibrium B content with SiC layer in the graphite crucible. Fundamental data for the successful use of Si-B alloys in the thermal energy storage system is needed and this paper will contribute by experimental data supported by theoretical calculations.

## 2. Materials and Methods

### 2.1. Phase Formation Experiments

**Figure 2:**
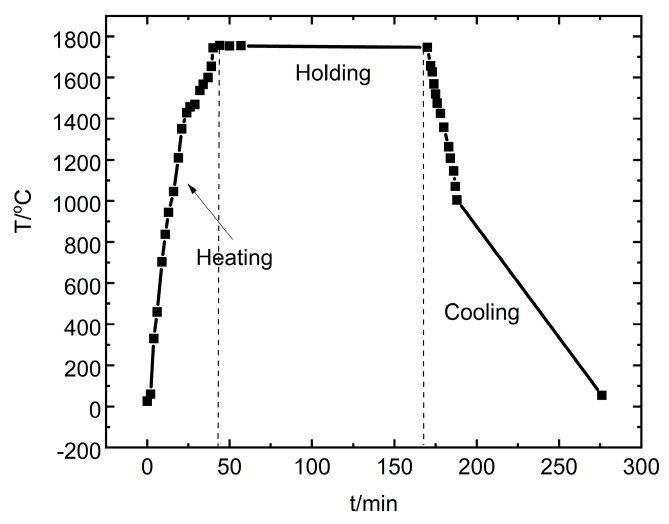
Typical temperature profile in the phase formation experiments. The black squares represent the measured temperature point.

In this study, Si-B alloys were produced by a mixture of electronic scale Si and 99.9 mass % B powder in a small graphite crucible with an outer diameter of 22 mm and an inner diameter of 18.5 mm. For simplicity, the electronic grade Si (SG-Si) was supplied from the Sintef industry (Trondheim, Norway), made from a fluidized bed reactor (FBR) process in the United States. In addition, 99.9 mass % B was supplied from Shanghai Aladdin Biochemical Technology Co., Ltd. (B-2, B105883, Shanghai, China). The graphite crucible (IG-15) is supplied by Svenska Tanso AB (Jönköping, Sweden). It is machined from a block of isostatically pressed graphite with several good properties, such as high reliability, ultra-heat resistance, good electrical conductivity, high thermal conductivity (140 W/m^−1^∙K^−1^), low porosity (10%), and good chemical resistance. In addition, the density of the graphite is 1.9 g/cm^3^. 2, 5, 8, and 11 mass % B powder was mixed with Si powder, respectively. Then, those Si-B mixtures were labeled as Si-2B, Si-5B, Si-8B, and Si-11B alloys. It aims to study the effect of B content on the phase formations in the Si-B alloys. The Si-B mixture was melted in a graphite crucible in an induction furnace at 1 bar argon atmosphere. Four different temperatures were chosen to study its effect on the phase formations, 1450, 1550, 1650, and 1750 °C. The holding time of 2 h at the holding temperature was included to facilitate the boron dissolution. In the end, the liquid Si-B alloys were cooled to room temperature with a cooling rate of 50 °C/min from max temperature to 1000 °C. The typical temperature profile is shown in Figure 2. It should be noted that a slow cooling rate would change the phase distribution during solidification.

**Figure 3:**
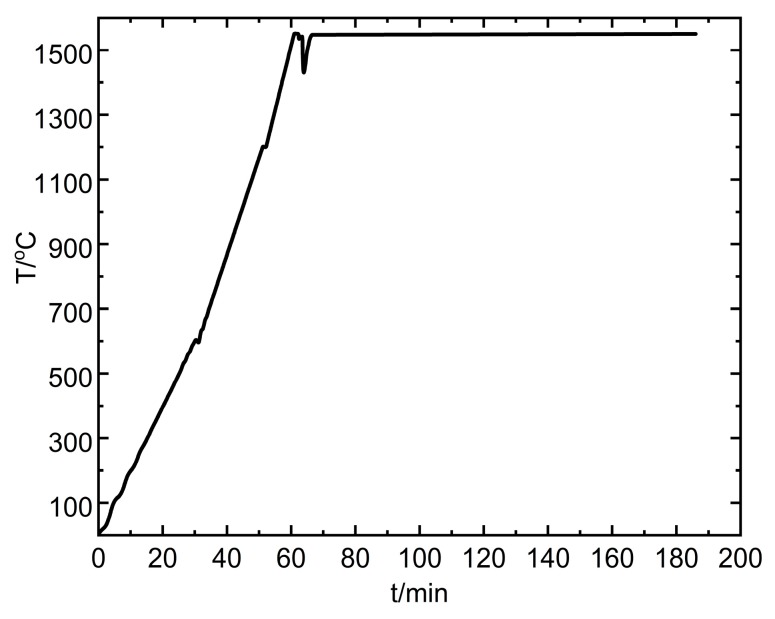
The temperature profile for the re-melt experiment in the resistance furnace, the peak at the plateau of 1550 °C represents the point that the charged crucible was placed in the center part of the furnace.

One experiment was conducted with the Si-B master alloy. The purpose of the experiment is to check the stability of the Si-B master alloy in the graphite crucible. The Si-5B master alloy was first produced in a graphite crucible. Then, several pieces of the Si-5B particles were placed in a new dense graphite crucible. The size of the crucible was the same as the one used in the phase formation experiments. Next, the charged crucible was heated in the resistance furnace under Ar and a holding time of 2 h was kept at 1550 °C in order to confirm the homogeneity of the molten Si-5B alloy. It should be noted that the furnace temperature was heated with an increase in the heating rate. When the furnace temperature reached 1550 °C, the charged crucible was placed in the center part of the furnace. Therefore, a sharp decrease in temperature happened at the plateau of 1550 °C. In the end, the furnace was shut off and the molten alloy was moved out of the furnace to make a fast solidification under Ar. The temperature profile of the heating part is shown in Figure 3. 

### 2.2. Carbon Solubility in the Molten Si-B Alloys

**Figure 4:**
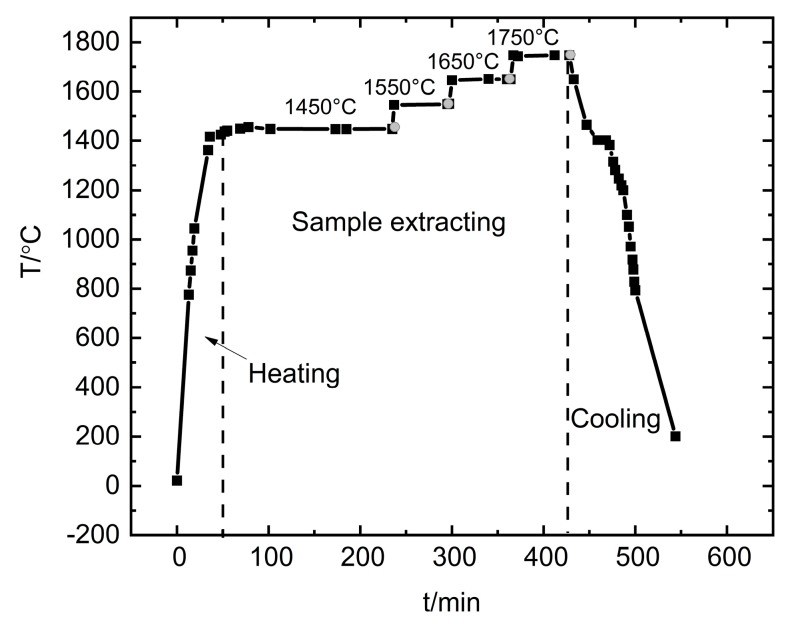
Typical temperature profile in the sample extraction experiments. The black square and grey circle represent the measured temperature points and extraction sample points.

The carbon solubility in the liquid Si-B alloys was investigated in the big graphite crucible with an outer diameter of 85 mm and 77.5 mm inner diameter under flow Ar. Si-B alloys of 2, 3.25, and 5 mass % of B were used. According to the Si-B binary phase diagram provided by Olesinski et al [6], Si-B alloys with the B addition of lower than 5 mass % would completely melt at 1450 °C. Hence, a holding time of 3 h at 1450 °C was used to facilitate the B dissolution. Then, a quartz tube was used to extract the molten Si-B sample at 1450 °C. It was fed into the furnace, down through the hole from the sampling window and into the molten Si-B alloys. Then, the C saturated molten Si-B alloys were sucked up by the use of a syringe. The quartz tube with 0.8–1.2 g of liquid sample was extracted. This process should be fast, as the quartz will be softened and can break at high temperatures. After the sample was extracted at 1450 °C, the molten Si-B was heated to 1550 °C within 2 min, continue to another 1 h holding time at this temperature, and then the extraction of the molten Si-B was performed at 1550 °C. The operation continued at 1650 °C and 1750 °C with the same procedures. The temperature profile for the Si-B samples extraction in one experimental run is shown in Figure 4. It is noted that the grey circles represent the extracting point. 

### 2.3. Microstructural Characterization

The samples should be treated before SEM observation. The samples were mounted in EpoFix cold-setting embedding resin (Agar Scientific, Essex, UK) and left for 12 h to harden. Then, these samples were prepared by an electronically controlled grinding and polishing machine. Next, the bottom and the edge of the sample were wrapped in tin foil. In the end, these samples were placed in a thermotank chamber, where the temperature was held at 100 °C, aiming to remove the residue gas and water from the sample.

Scanning electron microscope (SEM) (Zeiss Supra, 55 VP, Oberkochen, Germany) with a back-scatter-electron (BSE) detector was used for imaging. Phases were identified by using Energy Dispersive Spectroscopy (EDS). The Leco analyser (Leco CS-200, Mönchengladbach, Germany) was performed to analyze the carbon content in the Si-B alloys using an infrared combustion spectroscopy method. Moreover, the quantitative analysis of the extracted Si-B alloys was carried out by using ICP-HR-MS Agilent 8800™ (Santa Clara, CA, USA). It must be mentioned that to get the correct composition, the whole sample must be dissolved. This is also perhaps the main uncertainty of this process, as some black precipitate that can not be dissolved to the solution is found in the Si-B alloys, where the samples to be tested were digested in the 1.5 HCl + 0.5HF + 0.5HF acid. 

## 3. Results and Discussion

### 3.1. Phase Formation in C-Saturated Si-B Alloys

**Figure 5:**
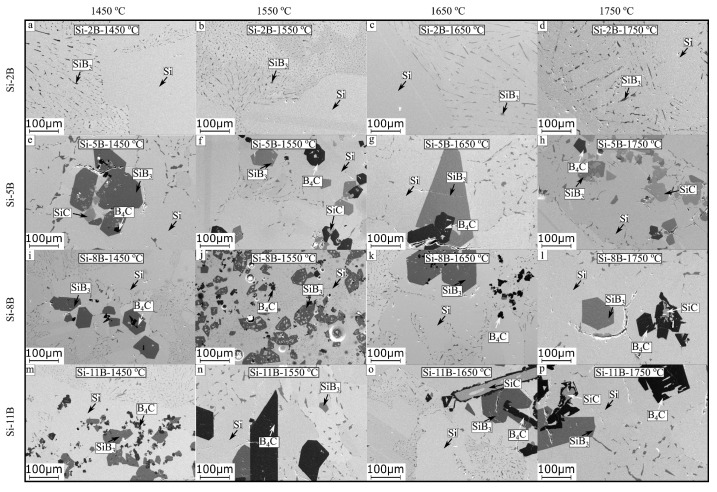
SEM images of the microstructures of samples Si-2B, Si-5B, Si-8B, and Si-11B alloys, keeping at 1450, 1550, 1650, and 1750 °C for 2 h. (**a**) Si-2B, 1450 °C; (**b**) Si-2B, 1550 °C; (**c**) Si-2B, 1650 °C; (**d**) Si-2B, 1750 °C; (**e**) Si-5B, 1450 °C; (**f**) Si-5B, 1550 °C; (**g**) Si-5B, 1650 °C; (**h**) Si-5B, 1750 °C; (**i**) Si-8B, 1450 °C; (**j**); Si-8B, 1550 °C; (**k**) Si-8B, 1650 °C; (**l**) Si-8B, 1750 °C; (**m**) Si-11B, 1450 °C; (**n**) Si-11B, 1550 °C; (**o**) Si-11B, 1650 °C; (**p**) Si-11B, 1750 °C.

The solidified Si-B alloys were cut with a diamond cut-off wheel. Then, it was performed by a stepwise electronically controlled grinding and polishing machine before SEM-EDS analysis. Figure 5a–p show the microstructures of Si-B alloys in the graphite crucible at 1450 °C, 1550 °C, 1650 °C, and 1750 °C. The original addition of B is 2, 5, 8, and 11 mass % in the Si-B alloys. It is found that the microstructures were simple in the Si-2B alloys based on the EDS point analysis, mainly Si(ss) and eutectic structure (Si + SiB_3_) were present (Figure 5a–d). However, a minor amount of B_4_C particles were also found in the extraction sample of the Si-2B alloy at 1450 °C (in Section 3.3). It shows that three different phases existed in the Si-2B alloys. Simultaneously, there were four different phases in the Si-5B, Si-8B, and Si-11B alloys. It includes Si(ss) (light grey regions), SiB_3_ (dark grey grains), B_4_C (dark grains), and SiC (grey grains) (Figure 5e–p). It is observed that SiB_3_ was in the form of either large grains or eutectic structures. Some SiC particles were surrounded by B_4_C (Figure 5f,l,o,p), some were formed inside the SiB_3_ large grains (Figure 5e,h), and others were isolated in the Si matrix (Figure 5e,f,h). The microstructures were determined by the B content in the Si-B alloys. In addition, the phases present did not vary much with varying temperatures at 1450–1750 °C.

**Figure 6:**
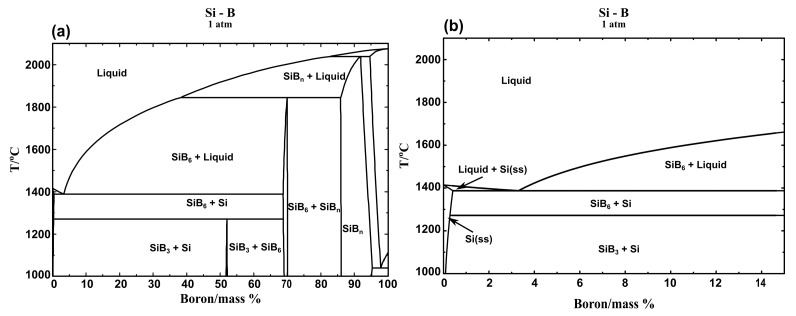
(**a**) isoplethal cross-section of the Si-B phase diagram; (**b**) enlarged part of the Si-B alloy in the B range 0–15 mass %. The phase diagrams were calculated with FactSage 7.2 in the pressure of 1 atm [22].

Figure 6a shows an isoplethal cross-section of the Si-B system. Figure 6b shows the enlarged part of the Si-B system in the B range 0-15 mass %. It is seen that the B content in the eutectic point is 3.25 mass %. Therefore, the Si-2B alloy is located at the hypoeutectic region. The microstructure in this region is characterized by primarily solidified Si surrounded by a eutectic structure (Figure 5a–d). With the decrease in temperature, the liquid Si-B alloy will have a eutectic reaction at 1385 °C, liquid → Si + SiB_6_. Then, the produced SiB_6_ is transformed to SiB_3_ at temperatures below 1270 °C, according to the reaction Si + SiB_6_ → SiB_3_. However, Si-5B, Si-8B, and Si-11B alloys are located at the hypereutectic region. It is clear that the primary solidified phase is SiB_6_ in the solidification process. The formed primary crystallized phase SiB_6_ acts as a nucleus at descending temperatures. Thus, the large grained SiB_6_ particles appear in the Si-B alloys at B content higher than 3.25 mass %. With the temperature decreasing, the formed SiB_6_ particle reacts with Si(ss) to form SiB_3_ with a peritectoid reaction. (Figure 5e–p). It shows a good agreement with our microstructural observation.

**Figure 7:**
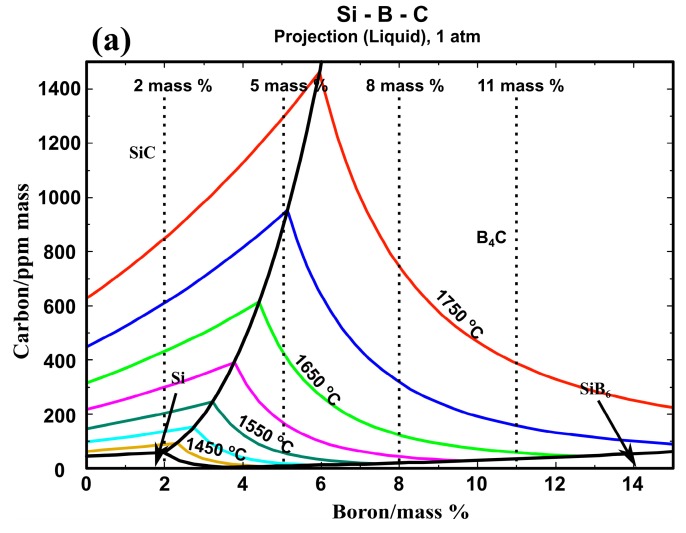

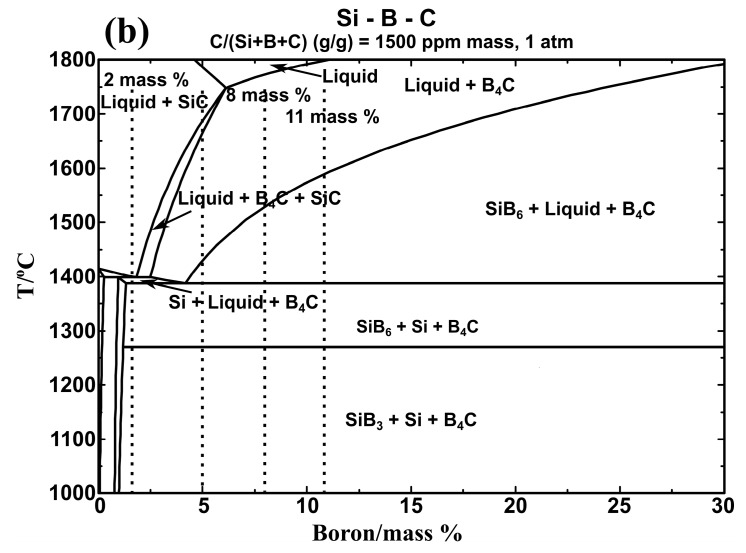
(**a**) liquidus projection of Si-B-C phase diagram calculated with FactSage 7.2; (**b**) isoplethal cross-section of the Si-B-0.15C (mass %) phase diagrams calculated with FactSage 7.2 [22].

However, the influence of C should be considered when the Si-B alloys are melted in the graphite crucible. In Figure 7a, the liquidus projection in the Si-B-C system was calculated. The dashed lines represent the Si-B alloys with the B contents of 2, 5, 8, and 11 mass %. As illustrated in Figure 7a, SiC coexists with molten Si-2B alloy at temperatures above 1450 °C and with molten Si-5B alloy at temperatures above 1700 °C. Otherwise, B_4_C equilibrates with molten Si-5B alloy at temperatures below 1700 °C and with molten Si-8B and Si-11B alloys above their melting point. The C saturation in the Si-B alloys is lower than 1450 ppmm at B contents lower than 15 mass % and temperatures below 1750 °C. Therefore, by assuming a C content above 1500 ppmm, the Si-B-C system will be saturated in C. Subsequently, the Si-B-0.15C (mass %) phase diagram was calculated, as shown in Figure 7b. It is seen that the Si-B alloys are located at the SiB_3_ + Si + B_4_C region at temperatures below 1270 °C in the B contents of 2–11 mass %.

**Figure 8:**
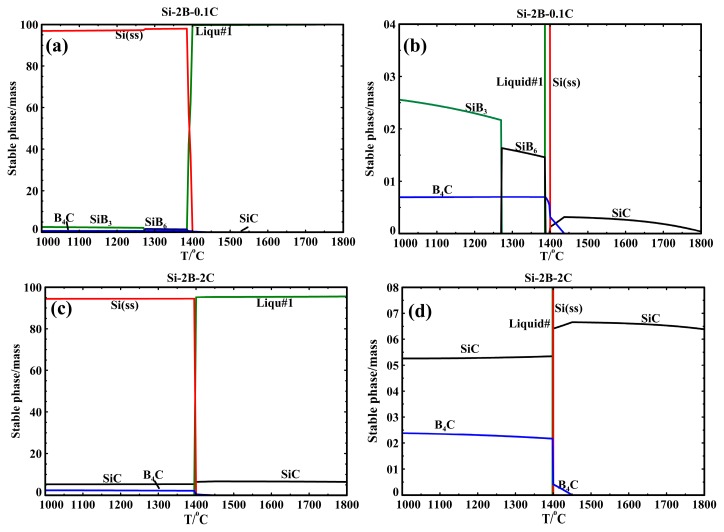
The phase evolution of the cooling process from 1800 °C, (**a**) Si-2B-0.1C; (**b**) magnification of the lower part in the Si-2B-0.1C system; (**c**) Si-2B-2C; (**d**) magnification of the lower part in the Si-2B-2C system [22].

In order to explain the influence of C content on the phase formation in the Si-B-C system, the thermodynamic modeling of phase equilibria was calculated with FactSage based on an FTlite database [22]. Figure 8 shows the phase evolution for the Si-2B alloys at the graphite crucible in the temperature range 1000–1800 °C. Two different conditions were considered in the calculation process, C content higher than the saturation level at 1750 °C (Figure 8c,d) and lower than the saturation level at 1750 °C (Figure 8a,b). At high C content, SiC would be in equilibrium with liquid alloy at high temperatures and SiC, B_4_C, and Si(ss) solid solution precipitated during cooling. As the B would be precipitated as B_4_C, there was no B left to form the SiB_3_ phase. At low C content, SiB_6_, Si(ss) and B_4_C were formed in the solidification process. SiB_6_ was transformed into SiB_3_ with a further decrease in the temperature. Therefore, the precipitated phases were B_4_C, SiB_3_, and Si(ss). Considering the cooling rate (Figure 2), it is not believed that the SiC particles can be completely transformed to B_4_C particles in the solidification process, leading to the SiB_3_ SiC, B_4_C, and Si(ss) four phases precipitated in the solidified Si-B alloys. In addition, it is possible that SiC was detached from the SiC interlayer, and then moved to the molten Si-B alloys during cooling process, leading to the SiC phase existed in the Si-B alloys. It is hence believed that at the holding temperature the alloy was C-saturated, but it may contain no solid phase in the bulk phase. The borides and the carbides would be precipitated during cooling. As no SiB_6_ phase was found, it is also seen that this phase is rapidly transformed to SiB_3_ during cooling.

### 3.2. Interlayer Phase Formation in the Si-B Alloys

**Figure 9:**
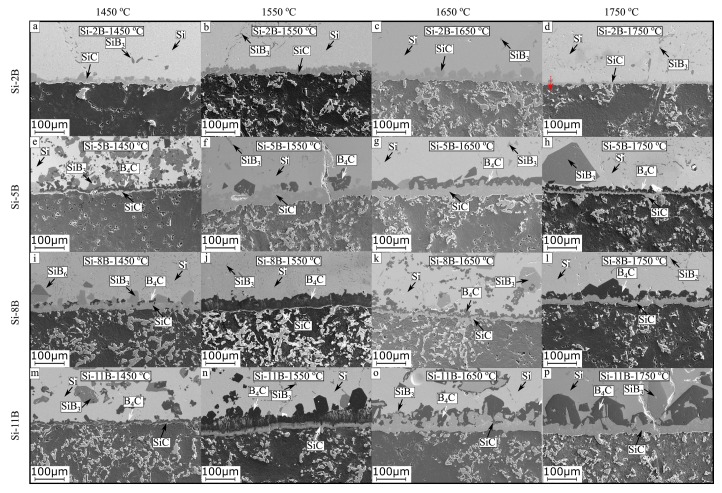
SEM images of the interlayer phase distribution between samples Si-2B, Si-5B, Si-8B, and Si-11B alloys and graphite, the samples were kept at 1450, 1550, 1650, and 1750 °C for 2 h, respectively. Then, these samples were cooled to room temperature (as shown in Figure 2). The red dotted arrow represents the EDS line scan position. (**a**) Si-2B, 1450 °C; (**b**) Si-2B, 1550 °C; (**c**) Si-2B, 1650 °C; (**d**) Si-2B, 1750 °C; (**e**) Si-5B, 1450 °C; (**f**) Si-5B, 1550 °C; (**g**) Si-5B, 1650 °C; (**h**) Si-5B, 1750 °C; (**i**) Si-8B, 1450 °C; (**j**); Si-8B, 1550 °C; (**k**) Si-8B, 1650 °C; (**l**) Si-8B, 1750 °C; (**m**) Si-11B, 1450 °C; (**n**) Si-11B, 1550 °C; (**o**) Si-11B, 1650 °C; (**p**) Si-11B, 1750 °C.

**Figure 10:**
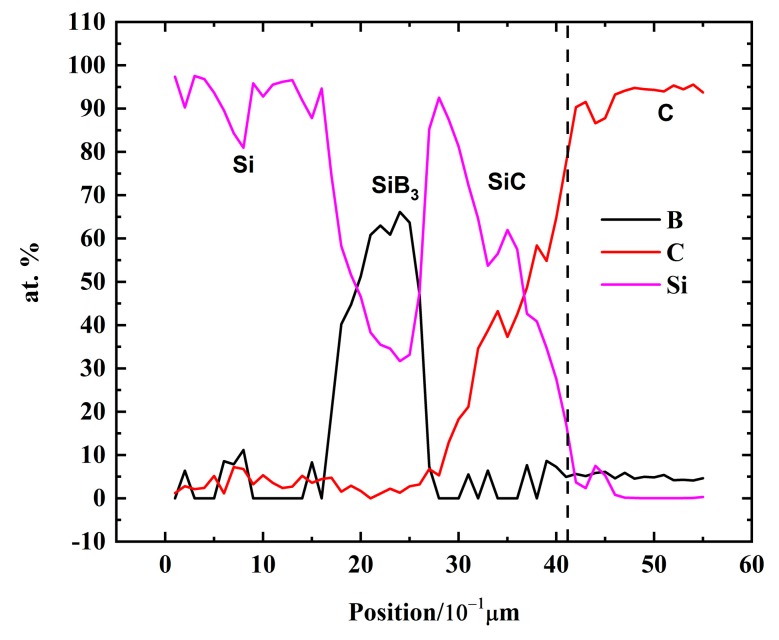
EDS line-scan of B, C, N, O, and Si across the interface between the Si-2B alloys and graphite at 1750 °C (red arrow in Figure 9).

Figure 9a–p show the cross-sectional SEM images for the interlayer phase distributions between the solidified Si-B alloys and graphite in the phase formation experiments. As seen inside the graphite, SiC particles were produced. Moreover, a small quantity of B_4_C particles was confirmed to be produced inside the graphite according to the line scan (Figure 10) where B was detected inside the graphite. It shows that the molten Si-B alloy penetrated the graphite to form SiC and B_4_C particles. At the interface, a continuous SiC layer was produced between the Si-2B alloy and graphite at temperatures 1450–1750 °C (Figure 9a–d). However, at B contents higher than 5 mass %, the interface was changed to two layers, SiC and B_4_C layers. The SiC layer close to the graphite was believed to be formed by the reaction between the liquid Si and graphite [25]. The B_4_C layer close to the Si-B alloys was formed by the reaction between B in the solution and the SiC layer, 4SiC(s) + 4B (l) → B_4_C(s) + 4Si(l). 

**Figure 11:**
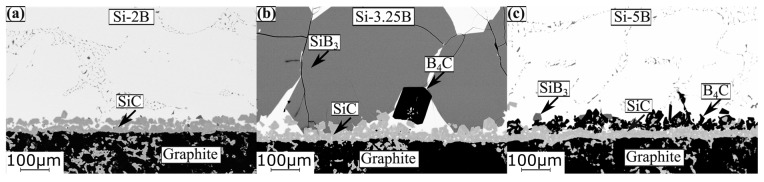
SEM images (BSE contrast) of the interlayer phase distributions between Si-B alloys and graphite in the extraction experiments. (**a**) Si-2B; (**b**) Si-3.25B; (**c**) Si-5B.

Figure 11 shows the BSE images of the produced interlayer phase after sample extractions from the graphite crucible in the temperatures range 1450–1750 °C. It is seen from the figure that SiC layer was formed between the Si-2B alloy and graphite, while SiC and B_4_C layers were produced between Si-5B alloy and graphite. It is in accordance with the interlayer phase distributions in the phase formation experiments. At the interlayer between Si-3.25B alloy and graphite, a continuous SiC layer was formed. Moreover, the large size of SiB_3_ particles was produced close to the SiC layer. The formed SiC layer in the Si-3.25B/graphite system was in good agreement with Grorud [26] that the Si-3.25B alloy was kept in the graphite crucible for 48 h at 1550 °C. Therefore, it is found that a single SiC layer was produced in the Si-2B and Si-3.25B alloys, while SiC and B_4_C layers were produced in the Si-B alloys with the B content higher than 5 mass %.

**Figure 12:**
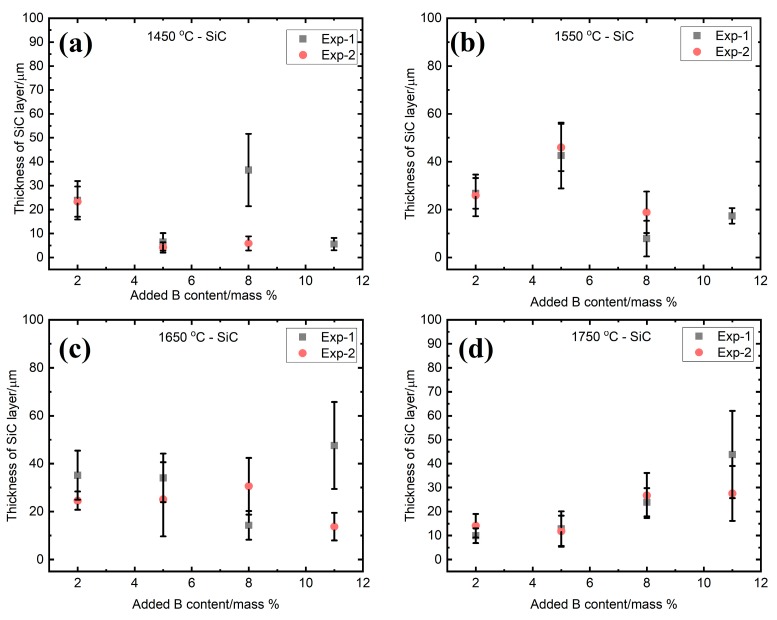
The thickness of SiC interlayer as a function of the B addition at four different temperatures. (**a**) 1450 °C; (**b**) 1550 °C; (**c**) 1650 °C; and (**d**) 1750 °C. Exp-1 and Exp-2 represent two different experiments in the same condition.

**Figure 13:**
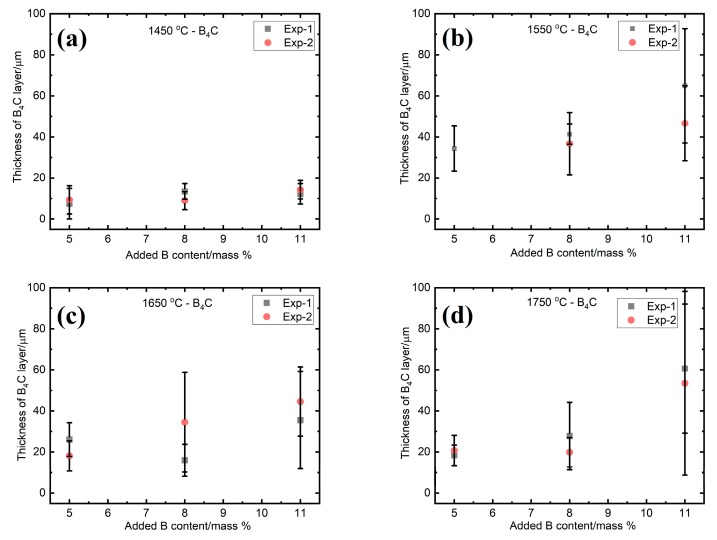
The thickness of the B_4_C interlayer as a function of the B addition at four different temperatures. (**a**) 1450 °C; (**b**) 1550 °C; (**c**) 1650 °C; and (**d**) 1750 °C. Exp-1 and Exp-2 represent two different experiments in the same condition.

**Figure 14:**
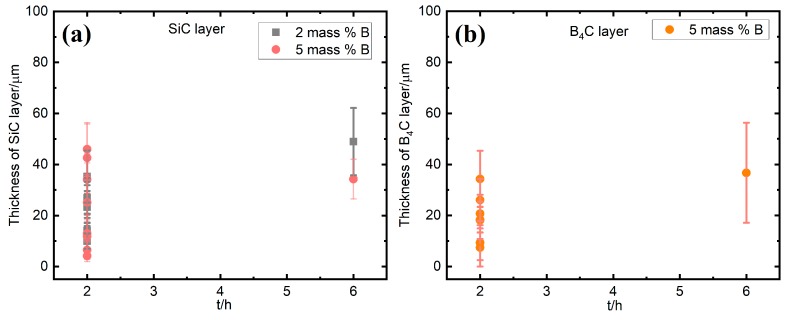
The thickness of the produced layer as a function of holding time at 1450–1750 °C; (**a**) SiC; (**b**) B_4_C.

The evolution of the average thickness of the SiC and B_4_C layers in the graphite crucible are shown in Figure 12, Figure 13 and Figure 14. The average values with the standard deviations are based on two experiments, labeled as Exp-1 and Exp-2. These experiments were done at the same conditions. It is seen from Figure 12 that the SiC layer in the small graphite crucible appeared to be thinning with the increasing of the B addition at 1450 °C (Figure 12a), while it remained nearly constant at 1550 °C and 1650 °C (Figure 12b,c), independent of the B addition. Once the temperature increased to 1750 °C, the SiC layer became thicker with B addition (Figure 12d). When it transformed to the B_4_C layer, it remained almost unchanged at 1450 °C, 1550 °C, and 1650 °C with the B addition (Figure 13a–c). At 1750 °C, the B_4_C layer did not significantly change at 5 and 8 mass % B, and it had an abrupt increase at 11 mass % B (Figure 13d). The measured thickness was in the range of 4–48 µm for the SiC layer, while it was 8–65 µm for the B_4_C layer. The standard deviation was caused by the nonuniformity of the layers. 

Figure 14 shows the thickness of SiC and B_4_C layer as a function of holding time at 1450–1750 °C. For Si-2B alloys, the thickness of SiC layer had a slight increase with the increasing of holding time. For the Si-5B alloys, both the thickness of SiC and B_4_C layers were in the same range after the holding time increased from 2 h to 6 h.

Grorud [26] investigated the interaction between the Si-3.25B alloy and graphite at 1450–1550 °C and the thickness of SiC was measured to be at the range of 10–80 µm after 1–48 h holding time. It is in accordance with our experimental results. 

**Figure 15:**
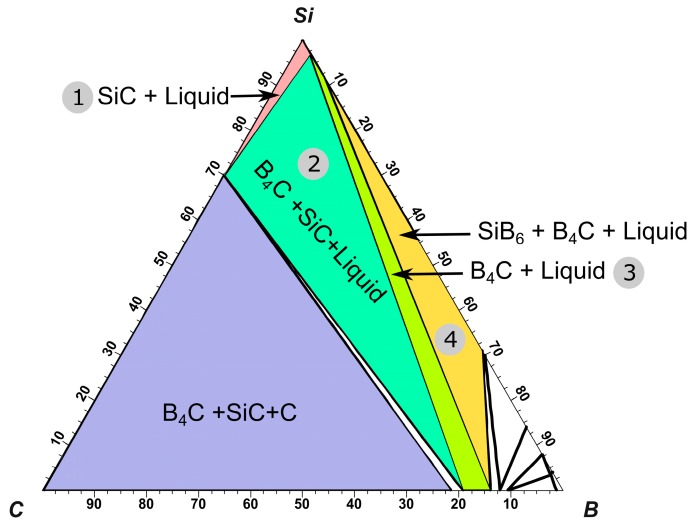
Isothermal section of the equilibrium phase diagram of the Si-B-C ternary system at 1550 °C (mass %) [22].

**Figure 16:**
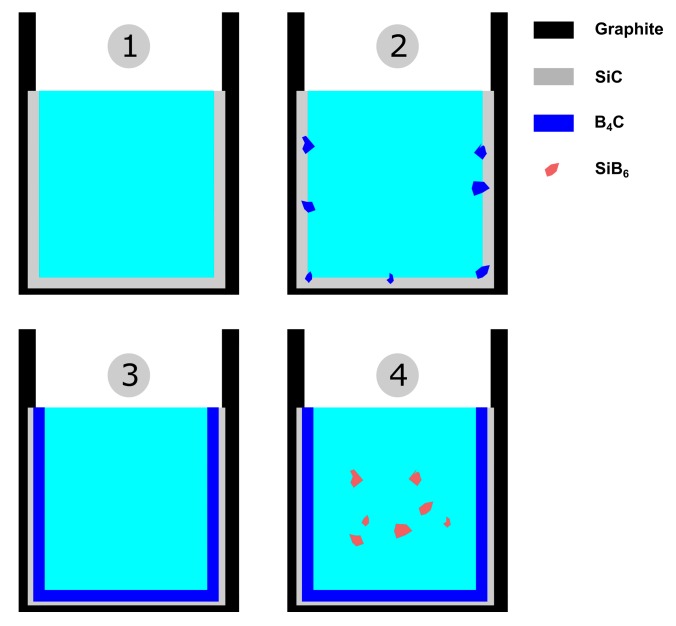
Illustration of the evolution of the reaction between molten Si-B alloys and graphite with the increase of B content at 1550 °C (1–4 correspond to the phase area in Figure 15)—1, 2, 3, and 4 correspond to the numbers in Figure 15.

Figure 15 shows the isothermal section of Si-B-C system at 1550 °C. The mechanism of the interface phase distribution is explained as follows. In the heating process, the molten Si-B alloy penetrates the graphite quickly, leading to B_4_C and SiC particles produced in the graphite, as shown in the purple area in Figure 15. Simultaneously, the SiC layer is formed between the molten Si-B alloy and graphite. Then, the system is changed to Si-B-SiC from Si-B-C. There are four different phase areas existing in the system based on the B content. At a low B content, the molten Si-B alloy equilibrates with SiC (Figure 16(1)). With the increasing B content, the system changes to the B_4_C + SiC + liquid area where liquid B starts to react with the SiC layer to form B_4_C (Figure 16(2)). Subsequently, the SiC layer is completely converted to B_4_C layer, and the molten Si-B alloy equilibrates with B_4_C layer (Figure 16(3)). If the B content continues to increase, SiB_6_ will be formed in the molten Si-B alloys (Figure 16(4)). Therefore, the B content determines the interlayer phase formation in the Si-B-C system.

As shown in the liquidus projection of Si-B-C phase diagram (Figure 7a), the black boundary line divides the SiC and B_4_C primary precipitation area. Therefore, it shows the B equilibrium content with SiC at different temperatures in the Si-B-C system. At B content of 2 mass %, the liquid Si-2B alloy and SiC is in thermodynamic equilibrium at temperatures over the melting temperature (~1400 °C). It supports the formation of SiC layer in the interface. At boron contents of 8 and 11 mass %, the molten Si-B alloy equilibrates with B_4_C at temperatures higher than their melting point. However, it changes to be more complicated at boron content of 5 mass %. Figure 7a shows that the liquid Si-5B alloy equilibrates with SiC at temperatures higher than 1700 °C, while it equilibrates with B_4_C below 1700 °C. It means that a phase transformation occurs from SiC to B_4_C at 1700 °C. Finally, the B_4_C layer is formed in the interface. At boron contents of 3.25 mass %, the liquid Si-3.25B alloy equilibrates with B_4_C at temperatures below 1550 °C. However, Grorud [26] and our present experiments show that SiC was the only phase formed at the interface at temperatures below 1750 °C. The thermodynamic calculation is inconsistent with the experimental results in the 3.25 and 5 mass % B alloys. The formation of the B_4_C layer at the alloy–container interface is depleting the B content in the liquid alloy phase. Therefore, further experiments should be performed to investigate the relationship between B content and the formation of B_4_C layer in the Si-B-C system.

### 3.3. The Determination of the Equilibrium B Content in the Si-B/Graphite System

As shown in Figure 7a and Figure 9a–p, the equilibrium B content in the liquid Si coexisting with the SiC interlayer should be lower than 5 mass %. Hence, Si-2B, Si-3.25B, and Si-5B alloys were chosen in the following high-temperature extraction experiments. The molten Si-B alloys were extracted quickly through quartz tubes at 1450, 1550, 1650, and 1750 °C, and then cooled fast to room temperature. 

**Figure 17:**
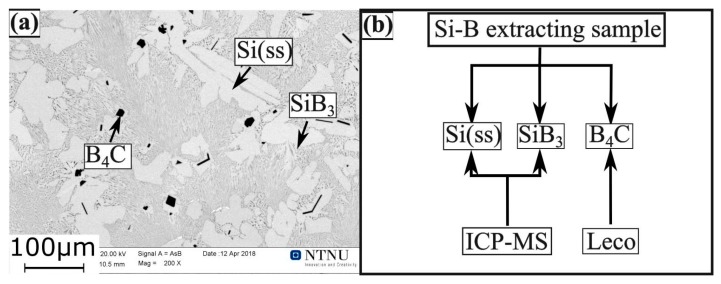
(**a**) SEM image (BSE contrast) of the microstructure of Si-2B extracting sample at 1450 °C; (**b**) calculation process for the B content in the extraction samples.

**Figure 18:**
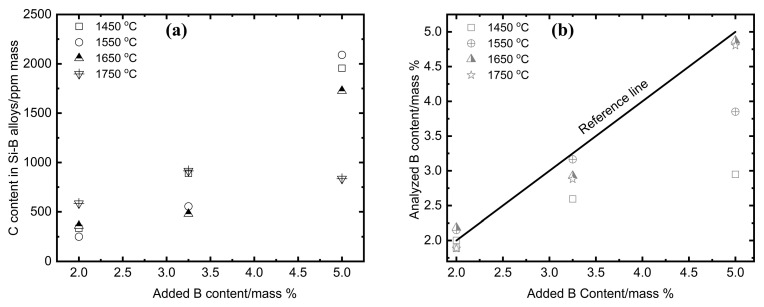
(**a**) C solubility in the Si-2B, Si-3.25B, and Si-5B alloys at 1450, 1550, 1650, and 1750 °C; (**b**) B content in the extraction Si-B samples (ICP-MS).

Figure 17a shows the microstructure of the extracted Si-2B sample after the holding time of 3h at 1450 °C. It is found that the liquid B was in the form of Si(ss), SiB_3_, and B_4_C in the solidified Si-B alloys. Additionally, B_4_C cannot be dissolved to the 1.5HNO_3_ + 0.5HF solution. Therefore, the retained B in the extracting samples can be calculated as for Figure 17b. The extracted samples were divided into two parts. One part was analyzed by Leco instrument to confirm the C content in the Si-B alloys with a parallel analysis (Figure 18a). Another part was analyzed by ICP-MS to determine its B content with a single analysis. The result is shown in Figure 18b.

**Figure 19:**
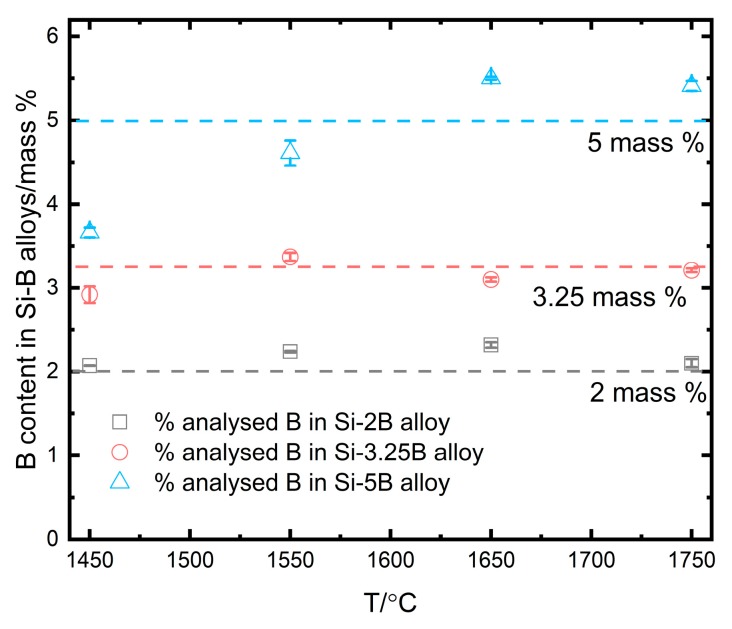
The left B content in extracted Si-B samples at different temperatures.

In the cooling process, the liquid C was in the form of Si(C) solid solution and B_4_C phase in the Si-B alloys. As the C solubility in the Si (C) solid solution was within 3–24 ppmm [27,28,29], the analyzed C content is assumed to be mainly from the B_4_C phase. Therefore, the B content (*C*_B1_) in the B_4_C phase can be determined by the analyzed C content. Simultaneously, the B content (*C*_B2_) in the Si(ss) and SiB_3_ phase was determined by ICP-MS. Hence, the retained B ((*C*_B_)) in the Si-B alloys was the sum of the *C*_B1_ and *C*_B2_, as shown in Figure 19.

It is seen from Figure 18a that the measured C content was in the range 250–585 ppmm in Si-2B melts, 480–915 ppmm in Si-3.25B melts, and 835–2090 ppmm in Si-5B melts. The carbon content increases with the B content in Si-B alloys. This is accordance with Halvor et al. [30] and Yanaba et al. [31]. In Figure 18b, the reference line represents the expected B content in the Si-B alloys that should be the same as the original added B content. The points represent the analyzed B content by ICP-MS. It corresponds well to the C content in the Si-B alloys (Figure 18a). The more C in the B_4_C particles, the less B in the SiB_3_ and Si(ss) phases. 

In Figure 19, the dotted lines represent the added boron content and the points with their standard deviation represent the residual B content in the extracted Si-B alloys after solidification. It is seen that the B loss is negligible in the Si-2B and Si-3.25B alloys at temperatures of 1450–1750 °C. However, the retained B contents is 3.66 mass % at 1450 °C, and 4.61 mass % at 1550 °C. They are lower than the added boron content of 5 mass %. It means that some B was depleting in the B_4_C layer in the Si-5B alloys at 1450 °C and 1550 °C. Hence, the B content of 3.66 mass % was regarded as the equilibrium B content in the reaction between molten Si-B alloys and SiC and B_4_C at 1450 °C. 

**Figure 20:**
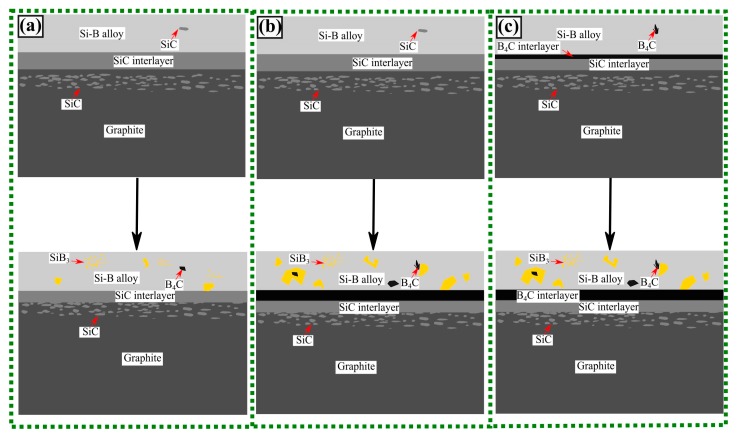
Illustration of the interaction of the graphite crucible and Si-B melt (**a**–**c**).

The following schema is proposed for the interaction of Si-B melts and graphite crucible (Figure 20). If the B content is lower than 3.66 mass %, the Si-B melts and SiC have a thermodynamic equilibrium at 1450–1750 °C (Figure 9a–d), and there is only a SiC layer produced between the Si-B alloy and graphite, as shown in Figure 20a. If the B content is higher than 3.66 mass %, there are two different situations. One is that the molten Si-B alloy equilibrates with SiC at high temperatures and the B_4_C layer was produced at the descending temperatures (Figure 9g,h). Another theory is that the molten Si-B alloy equilibrates with B_4_C in the whole temperatures range, as shown in Figure 20c.

### 3.4. Re-Melting of the Si-5B Master Alloy in Graphite Crucible

**Figure 21:**
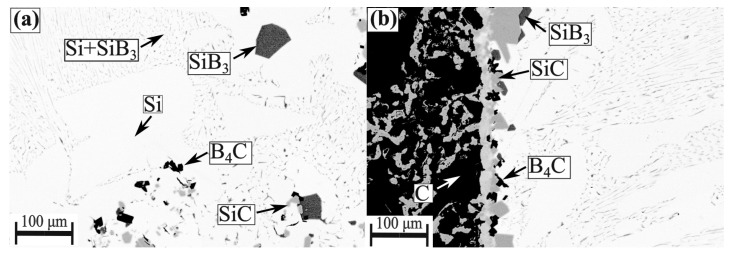
SEM images (BSE contrast) of the phase formation in the Si-5B alloy/graphite system. (**a**) in the center of the Si-5B master alloy; (**b**) at the interface between the Si-5B master alloy and graphite.

The Si-5B master alloy was produced in the sample extraction experiments in the temperature range 1450–1750 °C. After solidification, the B content was confirmed to be 3.48 mass % by ICP-MS. The C content was analyzed to be 835 ppmm at 1750 °C. Hence, the B content of the Si-5B master alloy was calculated to be ~4.095 mass % based on the method described earlier. It means that almost 1 mass % B was lost in the B_4_C interlayer between Si-5B alloy and graphite in the first melting time. Figure 21 shows the microstructures of the sample after re-melt of the Si-5B master alloy with the holding time of 2h at 1550 °C under Ar (now Si-4B) in the graphite crucible. It is seen from Figure 21a that Si, SiB_3_, B_4_C, and SiC phases were produced in the solidified alloy. It is consistent with the phases formed in the Si-5B alloys in the first melting time (Figure 5e–h). Figure 21b shows that some B_4_C particles were produced at the SiC interlayer. It confirms our calculation results that the B content higher than 3.66 mass % would produce B_4_C at the interlayer. In this regard, it is suggested that the Si-3.25B eutectic alloy might be the best suitable PCM to be used in the thermal energy storage systems. Moreover, its fusion enthalpy is calculated to be 1922 J/g (4.42 KJ/cm^3^) at 1385 °C [22]. 

The interaction of molten Si-3.25B alloys with graphite crucible was further investigated by Grorud [26] in our group. The molten Si-3.25B alloys were subjected to the repeated temperature cycles between 1430 °C and 1550 °C for 1–10 cycles or subjected to 1550 °C for 48 h. The results showed that the amounts of SiC particles in the Si-3.25B alloys were independent with the thermal cycles and holding times. Hence, it is possible to use dense graphite crucible as the refractory material for energy storage.

## 4. Conclusions

The present research was devoted to study the high-temperature interaction between Si-B alloys and graphite to find a suitable Si-B alloy as phase change material (PCM) and ultimately to decide if Si-B alloy in graphite crucible is suitable for the application in thermal energy storage (TES) system. The conclusions are summarized as follows.

Si-B alloys with various B contents (2–11 mass %) are melted in graphite crucible at varying temperatures from 1450–1750 °C. The molten Si-B alloys are carbon-saturated at high temperatures. Si solid solution, SiB_3_, B_4_C, and SiC are recognized after solidification. In addition, the amount of SiC particles in Si-B alloys is independent from the thermal cycles and holding times. It shows that the phases in the Si-B alloys are stable in the application of TES system.

A layer of SiC is produced along with the interface between Si-2B and Si-3.25B alloys and graphite. SiC and B_4_C layers are formed between Si-5B, Si-8B, and Si-11B alloys and graphite. The layers separate the Si-B alloys and graphite during melting/solidification cycles. SiC and B_4_C are found inside the graphite, and this means that a dense graphite container must be used in the TES system, as it shows a good wettability between the graphite and the alloy.

The carbon solubility is investigated in the Si-B alloys with the B addition of 2–5 mass % at varying temperatures from 1450–1750 °C. It is in the range 250–585 ppmm in molten Si-2B alloys, 480–915 ppmm in molten Si-3.25B alloys, and 835–2090 ppmm in molten Si-5B alloys. As the carbon content increasing with the B content in Si-B alloys, a higher B content Si-B alloy is not recommended to be used in the TES system.

The equilibrium B content is measured to be 3.66 mass % in the reaction between molten Si-B alloys and SiC at 1450 °C. Therefore, the B_4_C layer can be controlled based on the B addition in the Si-B alloys. Si-3.25B eutectic alloy is recommended as the new PCM in the graphite crucible.

## Figures and Tables

**Table 1:** Equilibrium reactions in the Si-B-C system at 1 atm [24].

T (°C)	Equilibrium Reaction
2385 (Point 1)	$liquid+C\to B_{4}C+\mathrm{SiC}$
2005 (Pont 2)	$liquid+B\to B_{4}{C+\mathrm{SiB}}_{n}$
1396 (Point 3)	$liquid+\mathrm{SiC}\to B_{4}C+\mathrm{Si}$
1850	$liquid{+\mathrm{SiB}}_{n}\to {\mathrm{SiB}}_{6}{+\;B}_{4}C$
1384	$liquid\to {\mathrm{Si}+\mathrm{SiB}}_{6}{+B}_{4}C$
1198	${\mathrm{Si}+\mathrm{SiB}}_{6}\to {\mathrm{SiB}}_{3}{+\;B}_{4}C$
